# Supplementary material for: Lag-invariant detection of interactions in spatially-extended systems using linear inverse modeling
Source: PLoS One. 2020 Dec 11;15(12):e0242715. doi: 10.1371/journal.pone.0242715 (PMC7732350; doi:10.1371/journal.pone.0242715)
Supplement: Attachment — Submitted filename: Hindriks_review.pdf [file pone.0242715.s014.pdf]

Hindriks review

In this paper, the author proposes a novel method to address the important problem of estimating phase-synchronization while accounting for spurious interactions due to MEG field spread or EEG volume conduction. The method is based on a subspace projection to suppress the contribution of source power to the sensor-space spectral matrix. In contrast to the original paper proposing this approach (Ossadtchi et al. (2018)), this paper demonstrates how it can be combined with any linear inverse operator to obtain a lag-independent estimator of system interactions. The method is novel and promising and the paper is well-written. However, a number of concerns should be resolved before the paper could be considered for publication:

Major concerns:

1. The method separates signal leakage-related and interaction-related contributions to sensor-space spectral matrix. However, it has been shown that the effect of signal leakage depends on the underlying true interaction strength (Fig. 3 in Palva et al. (2018)). In that sense, there is a portion of variance in the sensor-space spectral matrix that cannot be attributed to mere signal leakage or mere interaction but is due to the *combination* of the signal-leakage and underlying interaction strength. Does the method account for this?

   For example, consider interactions between two different pairs of signals. The source-power for both pairs of signals are the same, while source-coherence for the two pairs is different. Due to the differences in source-coherence, the effect of 'signal leakage' will also be different. Now, would this difference be reflected in the 'signal leakage' term? Alternatively, does this constitute a case where the 'source power' and 'source interactions' do not lie in different linear subspaces, as the method assumes?

   On a related note, I did not quite understand how the first term in equation 5 can have non-zero non-diagonal terms when in the previous line, it is mentioned the M can be split into M+ and M-, where M+ has all off-diagonal terms set to zero and M- has all diagonal terms set to zero. Given M+ has all off-diagonal terms set to zero, would we not expect the first term of equation 5 to also have all off-diagonal terms set to zero?

2. Related to point 1 above, how does the method's performance compare to obtaining corrected interaction strength by subtracting estimated interaction strength from interaction strength obtained from a surrogate dataset? The surrogate dataset is such that it has also been subject to MEG field spread, its 'source power' is the same as the original dataset but 'source interaction' is set to zero. Would the correction by the proposed method be essentially equivalent to this? It would be important to compare the method's performance with correction based on such a surrogate dataset.

3. The simulations based on two nodes are instructive and provide a basic demonstration of the method. However, since the assessment of the method is exclusively based on simulations, additional simulations should be done with more realistic simulated data. In particular, the correction should be applied to infer functional networks with *multiple* nodes. Between 80 and 120 nodes would make the network similar to those often estimated in MEG. The 'true' network should have interaction strengths ranging from 0 to 1 and the ability of the method to recover these strengths should be assessed. The amplitude and spectral properties of time series should also be similar to MEG data. For this, models of region dynamics such as Neural Mass Models (NMMs) (Jansen & Rit (1995)) could be used. Since properties of data and dimensionality of such a model would be similar to that encountered with MEG, good performance of the method on such a dataset would recommend its application to MEG.

4. The paper describes three variants of the proposed method. For a user of the method, are there any technical reasons for choosing one variant over another (apart from simulation results)? Does one method perform better than others in all cases? If not, in what situations should one use one variant over another? Clear description of technical differences between variants and guidance on when to use one variant over another would be useful. Alternatively, the paper could focus on a single variant if one variant performs best in all cases or if there are only small differences in performance between the variants.

5. All simulations were done with single examples. Where applicable, it would be good to perform simulations with multiple examples so that variability of method's performance can be assessed. In this regard, particularly for Figs 3-5, it would be good to see confidence intervals marked for all line plots.

6. Early in the paper, it would be good to have a simple diagram illustrating the method's operation, to complement the mathematical description.

7. In the Discussion, it would be good to describe any limitations of the method and particularly, in what practical situations might assumptions of the method be violated. Under what situations might errors be introduced into the estimation of corrected interaction strength (for e.g. low SNR? low lags?) How sensitive is the method to errors in the forward model? Further, how might the method work differently for EEG and for MEG data, and why? Some mention of these points is made while describing the method, but a fuller treatment in Discussion would be good. Any intuition on strengths, assumptions and limitations of the method would be very useful to future users.

8. A MATLAB and/or Python implementation of the proposed method could be made available to the community, in keeping with principles of Open Science.

Minor concerns:

1. Some typographical errors should be corrected:
   Page 3, final paragraph: "varies applications" should be "various applications"
   Page 10, final paragraph: "die to the mixing" should be "due to the mixing"
2. I might be mistaken, but in Page 17, would it not be that:
   T_imag=Imag(gamma(k,l))=abs(gamma(k,l))sin(theta(k,l)) , i.e. rather cos
   and
   T_real=Real(gamma(k,l))=abs(gamma(k,l))cos(theta(k,l)), i.e. rather than sin
3. Fig. 1B - z-axis labels should be specified
4. Fig. 2A - colour of lines for 'real' and 'imag' too similar, make colour of lines different and include legend
   Fig 2B - line colours and marker types too similar, change line colours and include legend
   Fig 2C - white dots showing true interaction could be change to bigger black dots for visibility, z-axis labels should be specified
   Fig. 2 caption - "strength of interaction wat set" should be "strength of interaction was set"
5. Fig. 3 - should include legend for black lines in A, B and C!
6. Fig. 4 - white dots showing true interactions could be bigger black dots for visibility, z-axis labels of images should be specified, colorbar scales could be equal to aid comparisons
7. Fig. 5 - x-axis labels could be 10 to 90, since 90 degrees is often mentioned in text. Panels A,C,E should be labelled 'Source-based' and changed to A,B,C. Panels B, D, F should be labelled 'Sensor-based' and changed to D,E,F.
